# Correlation Between Mask Compliance and COVID-19 Outcomes in Europe

**DOI:** 10.7759/cureus.24268

**Published:** 2022-04-19

**Authors:** Beny Spira

**Affiliations:** 1 Microbiology, Universidade de São Paulo, São Paulo, BRA

**Keywords:** mortality index, europe, linear correlation, masks, covid-19 transmission

## Abstract

Masking was the single most common non-pharmaceutical intervention in the course of the coronavirus disease 2019 (COVID-19) pandemic. Most countries have implemented recommendations or mandates regarding the use of masks in public spaces. The aim of this short study was to analyse the correlation between mask usage against morbidity and mortality rates in the 2020-2021 winter in Europe. Data from 35 European countries on morbidity, mortality, and mask usage during a six-month period were analysed and crossed. Mask usage was more homogeneous in Eastern Europe than in Western European countries. Spearman's correlation coefficients between mask usage and COVID-19 outcomes were either null or positive, depending on the subgroup of countries and type of outcome (cases or deaths). Positive correlations were stronger in Western than in Eastern European countries. These findings indicate that countries with high levels of mask compliance did not perform better than those with low mask usage.

## Introduction

Universal masking has been introduced during the coronavirus disease 2019 (COVID-19) pandemic at an unprecedented global scale as an important tool to curb viral transmission among potential susceptible persons. Face masks still are one of the most significant and controversial symbols in the fight against COVID-19. Two large randomised controlled trials about mask effectiveness performed during the pandemic came out with mixed results [1,2]. Several studies that analysed the effect of masks on the general population (ecological studies) have concluded that masks were associated with a reduction in transmission and cases [3-7]. However, these studies were restricted to the summer and early autumn of 2020. From March 2020 onwards, country after country instituted some form of mask mandate or recommendation. The stringency of these measures varied among the different countries and they, therefore, resulted in different proportions of mask compliance, ranging from 5% to 95% [8]. Such heterogeneity in mask usage among neighbouring countries provided an ideal opportunity to test the effect of this non-pharmaceutical intervention on the progression of a strong COVID-19 outburst.

## Materials and methods

Study design

This analysis aimed to verify whether mask usage was correlated with COVID-19 morbidity and mortality. Daily data on COVID-19 cases and deaths and on mask usage were obtained for all European countries. The rationale behind the choice of European countries for comparison was fourfold: (1) availability and reliability of data; (2) a relative population homogeneity and shared history of epidemics (comparing countries from different continents may bring too many confounding factors); (3) similar age stratification and access to health assistance; and (4) divergent masking policies and different percentages of mask usage among the different populations, despite the fact that the entire continent was undergoing an outburst of COVID-19 at the time period analysed in this study.

Inclusion criterion

Data were collected from the following Eastern and Western European countries: Albania, Bosnia and Herzegovina, Bulgaria, Croatia, Czechia, Hungary, North Macedonia, Poland, Romania, Serbia, Slovakia, Slovenia, Belarus, Estonia, Latvia, Lithuania, Republic of Moldova, Ukraine, Austria, Belgium, Denmark, Finland, France, Germany, Greece, Ireland, Italy, Netherlands, Norway, Portugal, Spain, Sweden, Switzerland, United Kingdom, and Northern Ireland. The inclusion criterion was a population size higher than one million people.

Data retrieval

Data on morbidity, mortality, and mask usage were retrieved from the Institute for Health Metrics and Evaluation (IHME) at the University of Washington [8]. Data from IHME were downloaded on 14th February 2022. IHME mask data sources are the Delphi Group at Carnegie Mellon University and the University of Maryland COVID-19 Trends and Impact Surveys, in partnership with Facebook, Kaiser Family Foundation, and YouGov COVID-19 Behaviour Tracker Survey (https://www.healthdata.org). Data on vaccination were obtained from Our World in Data (OWID) [9] on 4th April 2022.

Statistical analysis

Data from 35 European countries on morbidity, mortality, and mask usage during a six-month period were collected and analysed. Spearman’s correlation analyses and Shapiro-Wilk normality checks were in JASP (version 0.15; University of Amsterdam, Amsterdam, Netherlands) [10] and linear regressions in Wolfram Mathematica 13.0 (Wolfram Research, Inc., Champaign, Illinois) [11].

## Results

**Figure 1:**
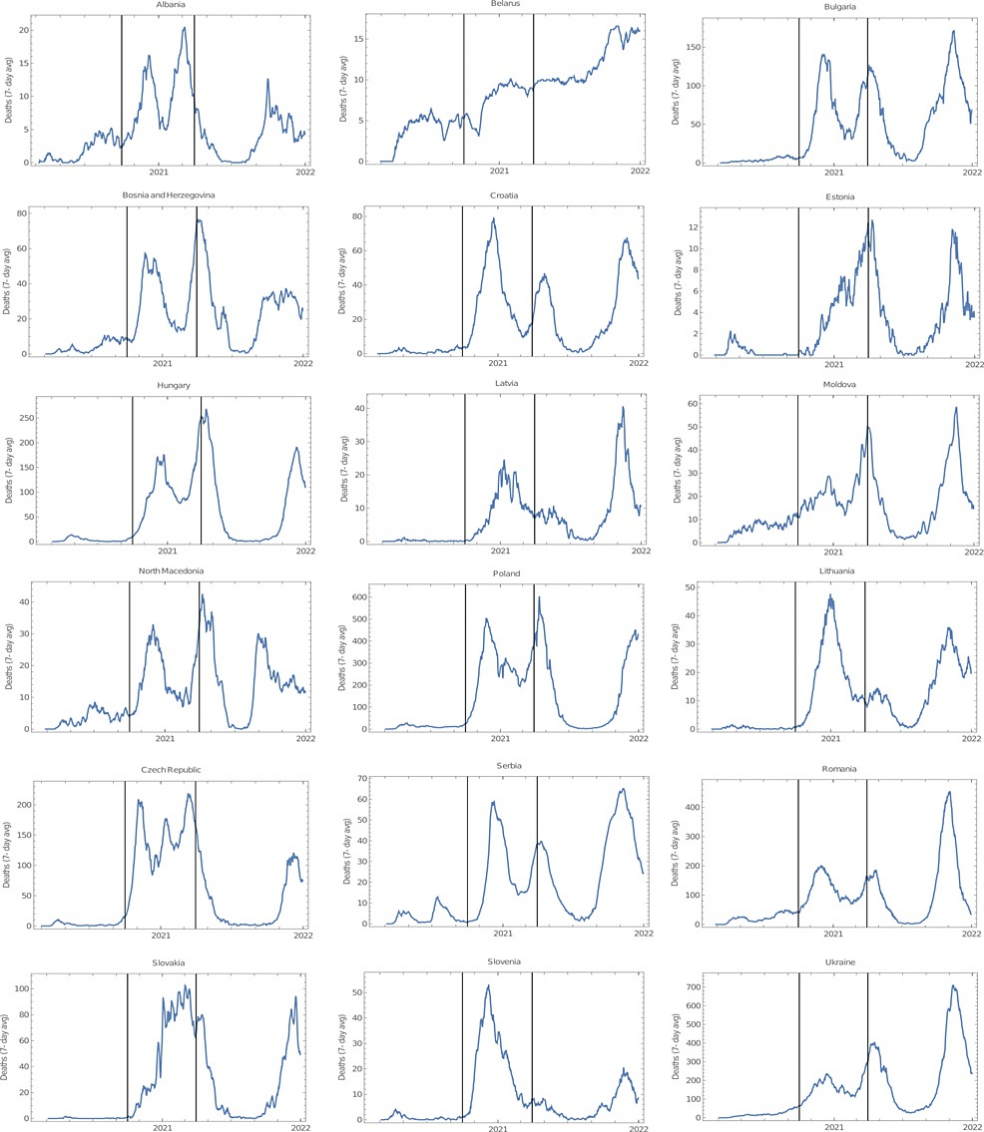
Mortality from COVID-19 throughout the pandemic in East European countries. The area between vertical black bars corresponds to the period analysed in this study (1 October 2020 to 31 March 2021). Data were downloaded on 14 February 2022 from Institute for Health Metrics and Evaluation (IHME).

**Figure 2:**
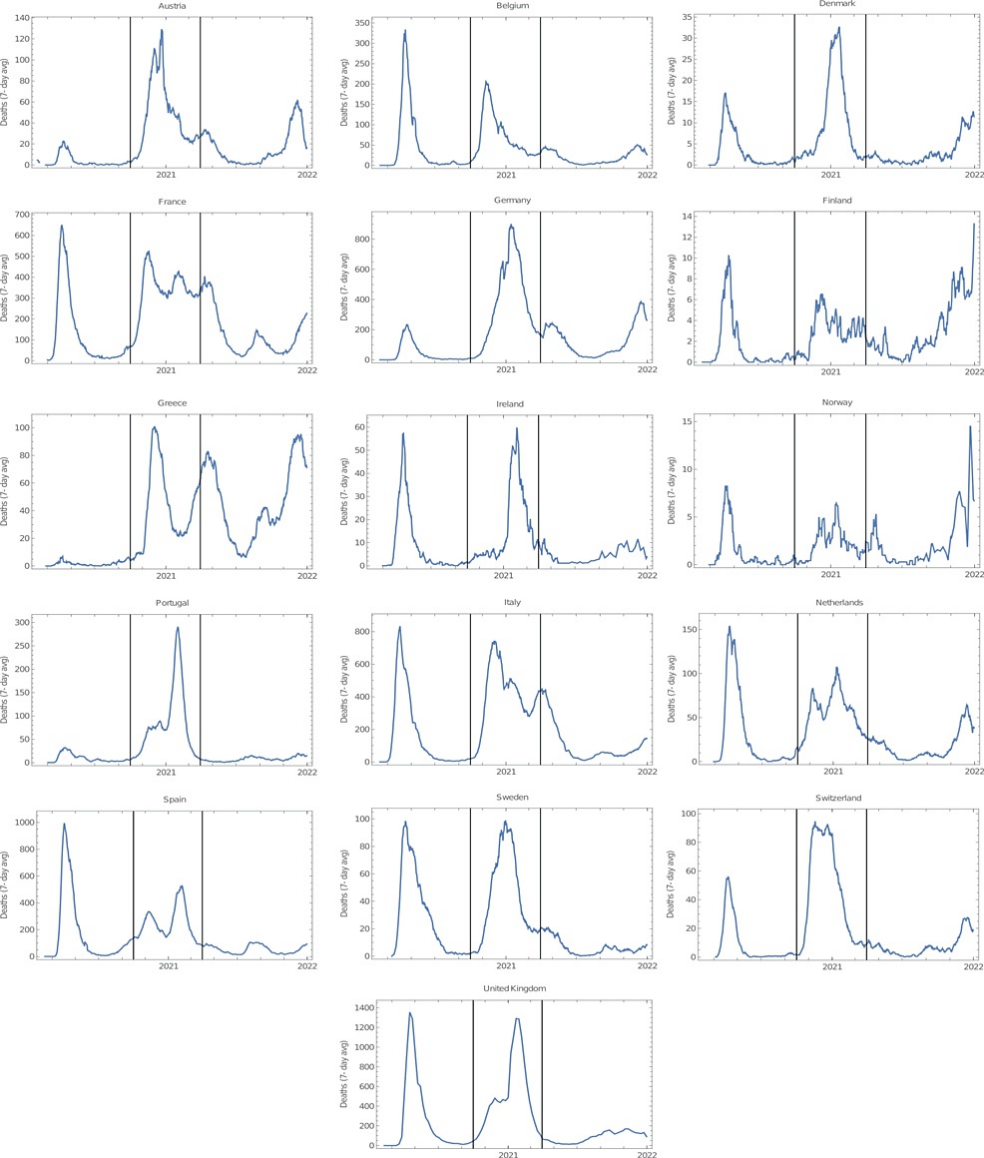
Mortality from COVID-19 throughout the pandemic in West European countries. The area between vertical black bars corresponds to the period analysed in this study (1 October 2020 to 31 March 2021). Data were downloaded on 14 February 2022 from Institute for Health Metrics and Evaluation (IHME).

This brief communication reports the correlation between the proportion of mask usage in the population and the number of cases (per million) and deaths (per million) from October 2020 to March 2021 in 35 European countries (Table 1). For this analysis, all European countries, including West and East Europe, with more than one million inhabitants were selected, encompassing a total of 602 million people. All analysed countries underwent a peak of COVID-19 infection during these six months (Figures 1, 2). The average proportion of mask usage in the referred period was 60.9% ± 19.9%, slightly higher in Eastern than in Western Europe (62.1% and 59.6%, respectively). However, the level of mask compliance was considerably more homogeneous in East (SD = 13.4%) than in West European countries (SD = 25.4%).

**Table 1:** Proportion of mask usage and the number of COVID-19 cases and deaths per million throughout the 2020-2021 late fall and winter (1st October to 31st March) in Europe. ^1 ^Percent of the population reporting always wearing a mask when leaving home. ^2 ^Shapiro-Wilk test for normality.

Country	Average mask usage^1^	Cases/million	Deaths/million
Albania	53%	40990	679
Bosnia and Herzegovina	40%	43078	1738
Bulgaria	55%	46405	1784
Croatia	29%	60039	1334
Czechia	52%	137494	2418
Hungary	77%	64704	2064
North Macedonia	67%	52048	1413
Poland	72%	57966	1315
Romania	81%	42898	1121
Serbia	54%	64829	521
Slovakia	76%	128326	1779
Slovenia	69%	101198	1879
Belarus	55%	25595	149
Estonia	64%	78525	639
Latvia	64%	52493	972
Lithuania	74%	75664	1252
Republic of Moldova	66%	48045	1102
Ukraine	67%	34298	686
Austria	55%	56237	959
Belgium	71%	66905	1135
Denmark	14%	34942	312
Finland	46%	12252	100
France	76%	58354	928
Germany	57%	29671	791
Greece	84%	23722	745
Ireland	71%	40270	587
Italy	91%	54310	1223
Netherlands	51%	68009	596
Norway	29%	15340	75
Portugal	84%	70056	1397
Spain	95%	55480	968
Sweden	5%	70356	759
Switzerland	53%	62669	927
United Kingdom	62%	57689	1363
Northern Ireland	68%	54567	1039
Shapiro-Wilk p-value^2^	0.056	0.004	0.693

**Figure 3:**
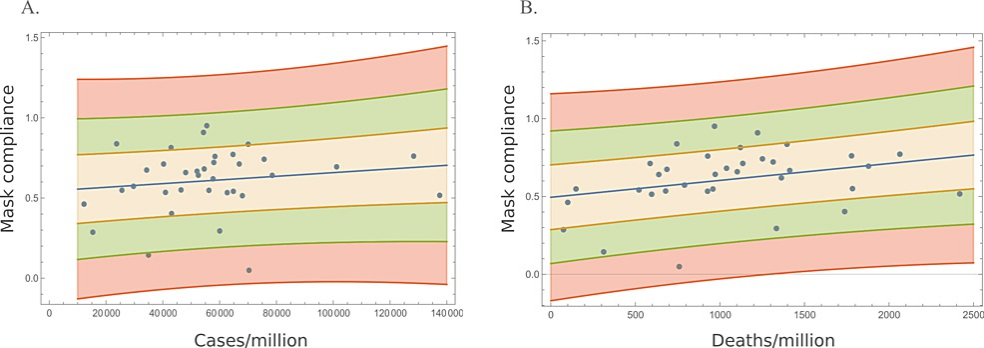
Correlation between average mask compliance and cases/million (A) or deaths/million (B) in 35 European countries. Each dot represents a country. The blue line represents the fitted regression line and the areas above and below indicate 1 $\sigma$ (yellow), 2 $\sigma$ (green), or 3 $\sigma$ (red).

Surprisingly, weak positive correlations were observed when mask compliance was plotted against morbidity (cases/million) or mortality (deaths/million) in each country (Figure 3). Neither the number of cases nor the proportion of mask usage followed a Gaussian distribution (Shapiro-Wilk p-values were 0.004 and 0.0536, respectively). A Spearman’s rank test was applied to quantify the correlation between mask usage, cases, and deaths (Table 2). The positive correlation between mask usage and cases was not statistically significant (rho = 0.136, p = 0.436), while the correlation between mask usage and deaths was positive and significant (rho = 0.351, p = 0.039). The Spearman’s correlation between masks and deaths was considerably higher in the West than in East European countries: 0.627 (p = 0.007) and 0.164 (p = 0.514), respectively. This difference could be associated with the fact that the most populous countries are located in West Europe. However, the correlations did not significantly change when the seven countries with populations > 20 million were excluded from the analysis (cases rho = 0.129 (p = 0.513); deaths rho = 0.375 (p = 0.049)). Analyses of other sub-groups, such as countries with populations smaller or higher than six million, higher than 10 million, or higher than 15 million, were also evaluated. None of these tests provided negative correlations between mask usage and cases/deaths.

**Table 2:** Spearman's rank correlation coefficient rho (p-value) between mask usage and COVID-19 cases or deaths. ^1^ Albania, Bosnia and Herzegovina, Bulgaria, Croatia, Czechia, Hungary, North Macedonia, Poland, Romania, Serbia, Slovakia, Slovenia, Belarus, Estonia, Latvia, Lithuania, Republic of Moldova, and Ukraine. ^2^ Austria, Belgium, Denmark, Finland, France, Germany, Greece, Ireland, Italy, Netherlands, Norway, Portugal, Spain, Sweden, Switzerland, United Kingdom, and Northern Ireland. * Statistically significant.

Territory	Masks x cases	Masks x deaths
All Europe	0.136 (0.436)	0.351 (0.039)*
Eastern Europe^1^	0.130 (0.606)	0.164 (0.514)
Western Europe^2^	0.05 (0.848)	0.627 (0.007)*

## Discussion

Mask mandates were implemented in almost all world countries and in most places where masks were not obligatory, their use in public spaces was recommended [12]. Accordingly, the World Health Organization (WHO) as well as other public institutions, such as the IHME, from which the data on mask compliance used in this study were obtained, strongly recommend the use of masks as a tool to curb COVID-19 transmission [8,13]. These mandates and recommendations took place despite the fact that most randomised controlled trials carried out before and during the COVID-19 pandemic concluded that the role of masks in preventing respiratory viral transmission was small, null, or inconclusive [1,2,14,15]. Conversely, ecological studies, performed during the first months of the pandemic, comparing countries, states, and provinces before and after the implementation of mask mandates almost unanimously concluded that masks reduced COVID-19 propagation [3-7,16]. However, mask mandates were normally implemented after the peak of COVID-19 cases in the first wave, which might have given the impression that the drop in the number of cases was caused by the increment in mask usage. For instance, the peak of cases in Germany's first wave occurred in the first week of April 2020, while masks became mandatory in all of Germany's federal states between the 20th and 29th of April [5], at a time when the propagation of COVID-19 was already declining. Furthermore, the mask mandate was still in place in the subsequent autumn-winter wave of 2020-2021, but it did not help preventing the outburst of cases and deaths in Germany that was several-fold more severe than in the first wave (Figure 2).

The findings presented in this short communication suggest that countries with high levels of mask compliance did not perform better than those with low mask usage in the six-month period that encompassed the second European wave of COVID-19. It could be argued that some confounding factors could have influenced these results. One of these factors could have been different vaccination rates among the studied countries. However, this is unlikely given the fact that at the end of the period analysed in this study (31th March 2021), vaccination rollout was still at its beginning, with only three countries displaying vaccination rates higher than 20%: the UK (48%), Serbia (35%), and Hungary (30%), with all doses counted individually [9]. It could also be claimed that the rise in infection levels prompted mask usage resulting in higher levels of masking in countries with already higher transmission rates. While this assertion is certainly true for some countries, several others with high infection rates, such as France, Germany, Italy, Portugal, and Spain had strict mask mandates in place since the first semester of 2020. In addition, during the six-month period covered by this study, all countries underwent a peak in COVID-19 infections (Figures 1, 2), thus all of them endured similar pressures that might have potentially influenced the level of mask usage.

## Conclusions

While no cause-effect conclusions could be inferred from this observational analysis, the lack of negative correlations between mask usage and COVID-19 cases and deaths suggest that the widespread use of masks at a time when an effective intervention was most needed, i.e., during the strong 2020-2021 autumn-winter peak, was not able to reduce COVID-19 transmission. Moreover, the moderate positive correlation between mask usage and deaths in Western Europe also suggests that the universal use of masks may have had harmful unintended consequences.
